# Supplementary material for: Human thalamic low-frequency oscillations correlate with expected value and outcomes during reinforcement learning
Source: Nat Commun. 2023 Oct 17;14:6534. doi: 10.1038/s41467-023-42380-6 (PMC10582006; doi:10.1038/s41467-023-42380-6)
Supplement: Supplementary file 1 — Supplementary information [file 41467_2023_42380_MOESM1_ESM.pdf]

Supplementary information

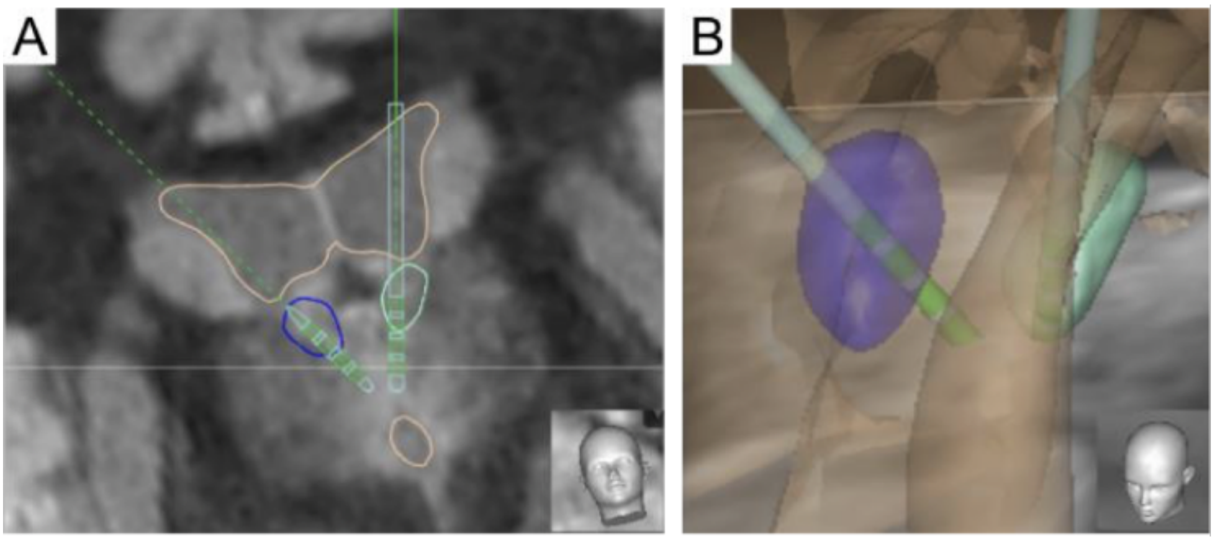

Figure S1. Anatomical location of thalamic electrodes. Reconstruction of intra-thalamic contact locations using intraoperative X-ray image coordinates, overlaid onto a preoperative FGATIR MRI mask. Representative data from a single patient: the upper contacts are situated within the anterior thalamic nucleus (ATN) as segmented by the neurosurgeon, while the lower contacts are positioned in the dorsomedial thalamic nucleus (DMTN). The ventricular system/third ventricle is depicted in orange, the ATN is shown in blue (right) and green (left), the electrodes are displayed in gray, and their trajectories are represented by dotted green lines. (A) MRI view of the nucleus implantation, aligned with the electrode orientation. (B) 3D reconstruction of the electrode within the nucleus.

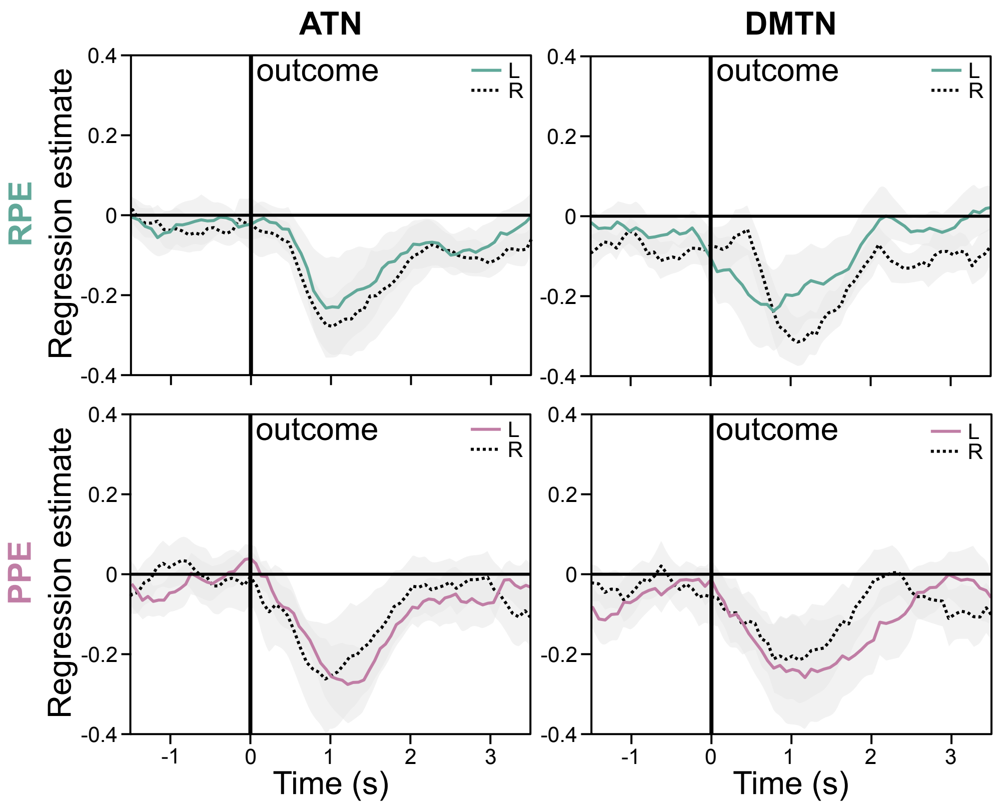

Figure S2. Reward and punishment prediction error signals in the left (L) and right (R) ATN and DMTN. Time course of regression estimates obtained from linear fit of low frequency oscillations (4-12 Hz) with prediction errors modeled separately for the reward and punishment conditions. PPE: punishment prediction error; RPE: reward prediction error. Grey shaded areas represent inter-sites SEM (n=24 sites). For both structures, no significant cluster (cluster-corrected, $p_c < 0.05$) in the time domain was found for the two-way Anova comparing the regression estimates in the structures (ATN, DMTN) and the sides (Left, Right) with interaction. Source data are provided as a Source Data file.

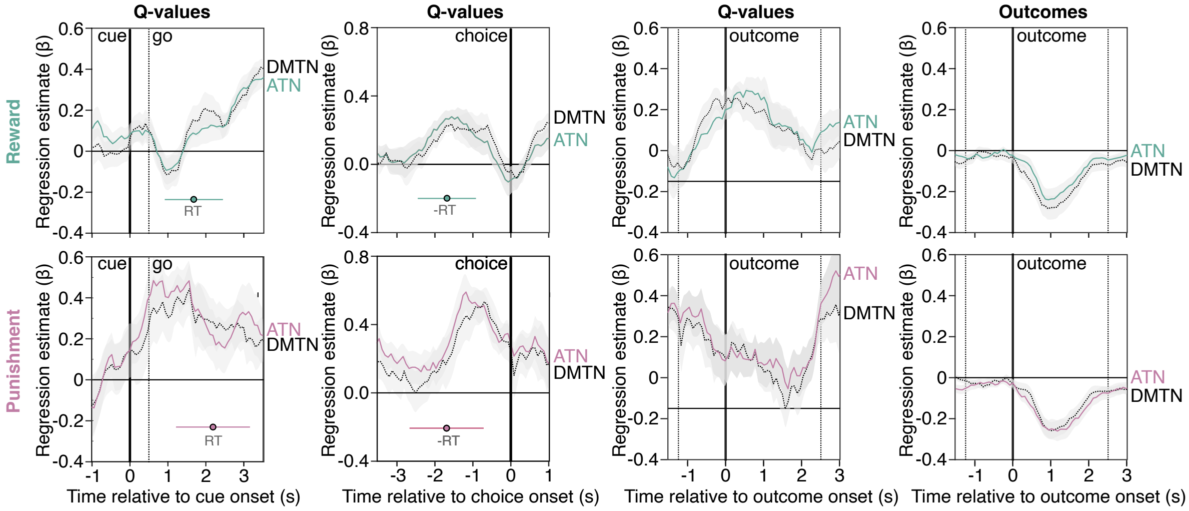

Figure S3. Comparison of low-frequency encoding of expectation and outcome between ATN and DMTN. Time-course regression in the 4-12 Hz frequency range with Q-values (Q_r, Q_p) and outcome (R, P) values with regression estimate averaged±SEM (the shaded gray area around the mean) across recording sites plotted separately for ATN (colored, n = 16 sites) and DMTN (black and dashed, n = 16 sites) in the punishment and reward conditions. No significant cluster (cluster-corrected, $p_c < 0.05$) in the time domain was found for the paired t-test comparing the regression estimates in the ATN and DMTN. Source data are provided as a Source Data file.

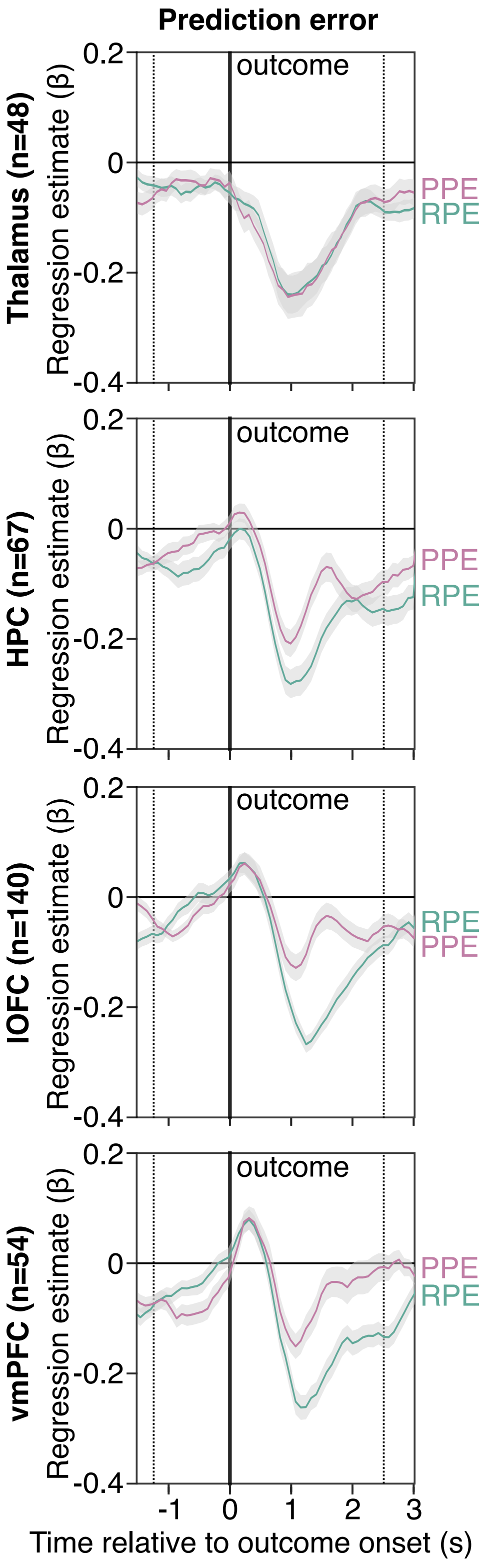

Figure S4. Comparison of LFO association with prediction errors in the thalamus (n=8 patients) and in the cortex (n=19 patients from Gueguen et al., 2021). Time-course regression in the 4-12 Hz frequency range with prediction error in the reward (RPE, green) and punishment (PPE, red) with regression estimate averaged±SEM (shaded gray area around the mean) across recording site plotted separately for thalamus (n = 48 sites, data from this study), hippocampus (HPC, n=67), lateral orbitofrontal cortex (lOFC, n=140), and ventromedial prefrontal cortex (vmPFC, n=54) (re-analysis of cortical data from Gueguen et al. 2022). Source data are provided as a Source Data file.

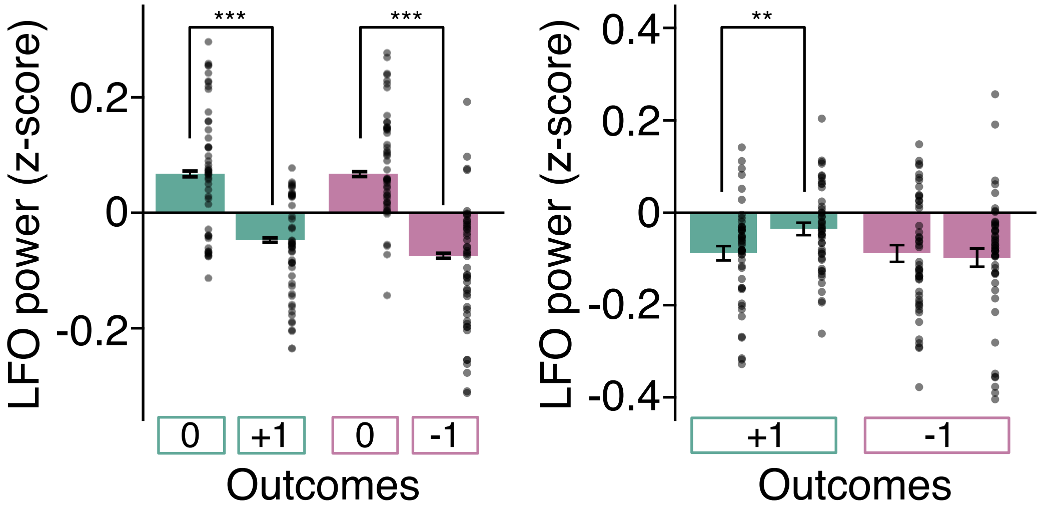

Figure S5. Average across thalamic LFO site at outcome onset (0-2 s). a. Average across thalamic sites (±SEM) LFO power in the 0-2s window following the outcome for each outcome in reward (green) and punishment (red) conditions. A two-sided paired t-test was performed to compare LFO power in the reward and punishment conditions revealed a significant difference between outcomes in both the reward condition ($t_{(47)}$ = 4.77, p-value = 1.85e-05) and the punishment condition ($t_{(47)}$ = 6.3161, p-value = 8.93e-08). **b.** Post-outcome decrease of LFO power is modulated by the level of expectation in the reward condition (green, median split on Q_r) but not in the punishment condition (red, median split on Q_p). Average across thalamic sites (±SEM) LFO power in the 0-2s window following the outcome onset separately estimated for low or high expectation trials (median split done separately for each subject and separately for the reward and punishment condition). A two tailed paired t-test was performed to compare LFO power between high and low expectation trials in the reward and punishment conditions revealed a significant difference between power in high vs. low expectation trials in the rewarding trials ($t_{(47)}$ = -3.16, p-value = 0.0028) but not in the punishing conditions ($t_{(47)}$ = 0.38, p-value = 0.71). Source data are provided as a Source Data file.

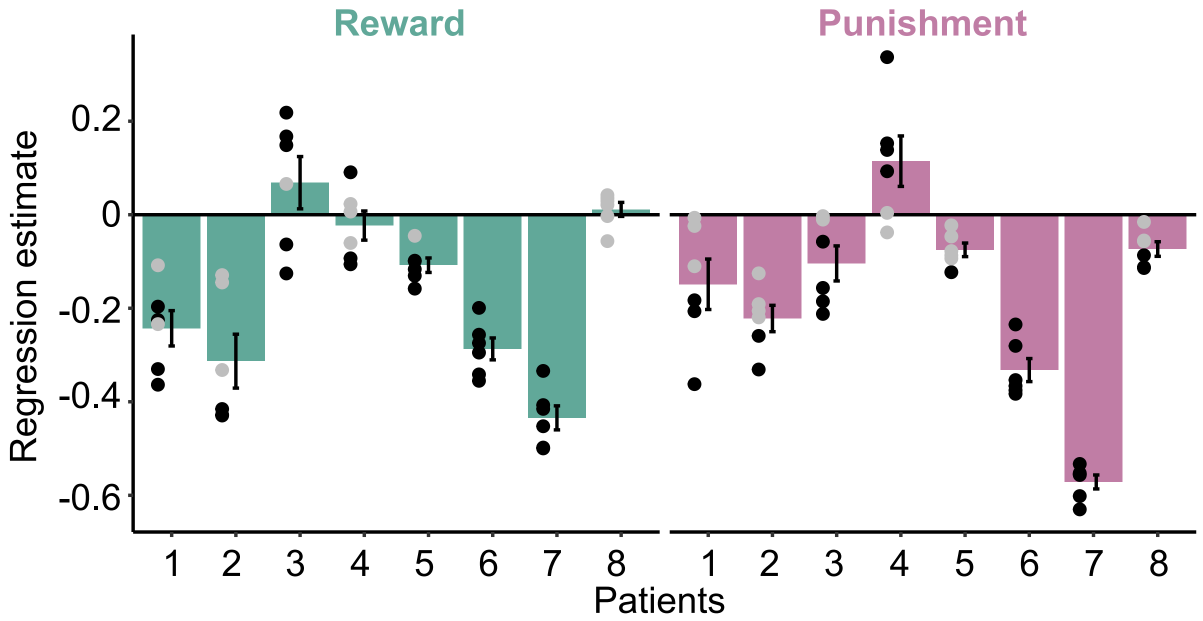

Figure S6. Individual electrophysiological data. Individual average±SEM across recording sites (n=48 sites) of regression estimates of prediction error against LFO power in the 0-2s window following the outcome in the reward and punishment conditions. Dots represent each recording site (n=6 per patient and condition), black (grey) dots corresponding to a recording site with a (un)significant regression (see Methods). Source data are provided as a Source Data file.

Table S1. Cohort data

Inclusion center	Age at first seizure (years)	Epileptic syndrome	Seizure origin	Medication at surgery	Number of sessions of 96 trials
Grenoble	<1	Frontal lobe epilepsy	bilateral	OXC, LAC, CLB, INN	6
Grenoble	27	Frontal lobe epilepsy	bilateral	LAC, CBZ, PRG	6
Grenoble	5	Temporo-occipital lobe epilepsy	bilateral	CLB, LZP, FBM	6
Grenoble	<1	Hypothalamic hamartoma	bilateral	LAC, RFN, CLB	6
Grenoble	7	Parietal lobe epilepsy	right	FBM, LEV, VGT, CLN	5
Grenoble	12	Occipital lobe epilepsy	bilateral	VPA, LTG, CLB	6
Marseille	32	Temporo-frontal lobe epilepsy	bilateral	CBZ, LAC, LEV, PGB, CLB	5
Marseille	7	Multifocal epilepsy	bilateral	OXC, TPM, CLB	4

OXC: oxcarbazepine; LAC: lacosamide; CLN: clonazepam; CLB: clobazam; INN: retigabine; CBZ: carbamazepine; PRG: pregabaline; LZP: lorapezam; FBM: felbamate; RFN: rufinamide; LEV: levetiracetam; VGT: vigabatrin; VPA: valproic acid; LTG: lamotrigine; PGB: pregabaline; TPM: topiramate
