# Supplementary material for: Human thalamic low-frequency oscillations correlate with expected value and outcomes during reinforcement learning
Source: Nat Commun. 2023 Oct 17;14:6534. doi: 10.1038/s41467-023-42380-6 (PMC10582006; doi:10.1038/s41467-023-42380-6)
Supplement: Supplementary file 3 — Reporting Summary [file 41467_2023_42380_MOESM3_ESM.pdf]

nature portfolio

Corresponding author(s): Julien Bastin

Last updated by author(s): Jul 11, 2023

Reporting Summary

Nature Portfolio wishes to improve the reproducibility of the work that we publish. This form provides structure for consistency and transparency in reporting. For further information on Nature Portfolio policies, see our Editorial Policies and the Editorial Policy Checklist.

Statistics

For all statistical analyses, confirm that the following items are present in the figure legend, table legend, main text, or Methods section.

n/a	Confirmed	
☐	☒	The exact sample size (*n*) for each experimental group/condition, given as a discrete number and unit of measurement
☐	☒	A statement on whether measurements were taken from distinct samples or whether the same sample was measured repeatedly
☐	☒	The statistical test(s) used AND whether they are one- or two-sided<br>*Only common tests should be described solely by name; describe more complex techniques in the Methods section.*
☐	☒	A description of all covariates tested
☐	☒	A description of any assumptions or corrections, such as tests of normality and adjustment for multiple comparisons
☐	☒	A full description of the statistical parameters including central tendency (e.g. means) or other basic estimates (e.g. regression coefficient) AND variation (e.g. standard deviation) or associated estimates of uncertainty (e.g. confidence intervals)
☐	☒	For null hypothesis testing, the test statistic (e.g. *F*, *t*, *r*) with confidence intervals, effect sizes, degrees of freedom and *P* value noted<br>*Give P values as exact values whenever suitable.*
☒	☐	For Bayesian analysis, information on the choice of priors and Markov chain Monte Carlo settings
☒	☐	For hierarchical and complex designs, identification of the appropriate level for tests and full reporting of outcomes
☐	☒	Estimates of effect sizes (e.g. Cohen's *d*, Pearson's *r*), indicating how they were calculated

Our web collection on statistics for biologists contains articles on many of the points above.

Software and code

Policy information about availability of computer code

Data collection	iEEG data were collected with Micromed (Treviso, Italia). Behavioral data were collected with Presentation (v16.5).
Data analysis	Behavioral data were analyzed with Matlab 2016. iEEG data were analyzed with Matlab 2016, FieldTrip (version r7276). All scripts are available upon request. Details are provided in Methods.

For manuscripts utilizing custom algorithms or software that are central to the research but not yet described in published literature, software must be made available to editors and reviewers. We strongly encourage code deposition in a community repository (e.g. GitHub). See the Nature Portfolio guidelines for submitting code & software for further information.

Data

Policy information about availability of data

All manuscripts must include a data availability statement. This statement should provide the following information, where applicable:

- Accession codes, unique identifiers, or web links for publicly available datasets
- A description of any restrictions on data availability
- For clinical datasets or third party data, please ensure that the statement adheres to our policy

The behavior and neural data that support the findings of this study are available in the figshare repository: https://figshare.com/s/4e30fed3055c02f37e2a

Research involving human participants, their data, or biological material

Policy information about studies with human participants or human data. See also policy information about sex, gender (identity/presentation), and sexual orientation and race, ethnicity and racism.

Reporting on sex and gender	8 patients, 3 females
Reporting on race, ethnicity, or other socially relevant groupings	*Please specify the socially constructed or socially relevant categorization variable(s) used in your manuscript and explain why they were used. Please note that such variables should not be used as proxies for other socially constructed/relevant variables (for example, race or ethnicity should not be used as a proxy for socioeconomic status).*<br>*Provide clear definitions of the relevant terms used, how they were provided (by the participants/respondents, the researchers, or third parties), and the method(s) used to classify people into the different categories (e.g. self-report, census or administrative data, social media data, etc.)*<br>*Please provide details about how you controlled for confounding variables in your analyses.*
Population characteristics	8 patients (38.1 ± 3.9 years old, 3 females) suffering from drug-resistant focal epilepsy
Recruitment	Participants were automatically involved in our study few days after the electrode implantation.<br>The access to the patient was granted by an ethics committee as described below.
Ethics oversight	The study received approval from the Institutional Review Board and by the National French Science Ethical Committee (Comité de Protection des Personnes Sud-Est I, protocol number: 2011-A00083-38).

Note that full information on the approval of the study protocol must also be provided in the manuscript.

Field-specific reporting

Please select the one below that is the best fit for your research. If you are not sure, read the appropriate sections before making your selection.

☒ Life sciences ☐ Behavioural & social sciences ☐ Ecological, evolutionary & environmental sciences

For a reference copy of the document with all sections, see nature.com/documents/nr-reporting-summary-flat.pdf

Life sciences study design

All studies must disclose on these points even when the disclosure is negative.

Sample size	We collected iEEG data in as many patients as we could. The main analyses are conducted across 48 data points (electrode contact-pairs) recorded across 8 patients. Thus, we consider our sample size as sufficient to investigate the effects we observe.
Data exclusions	Following Reviewer 1's comment, we excluded trials containing suspicious epileptiform neural activity (see Methods for more details).
Replication	The iEEG data are a rare dataset. Consequently, we could not provide a replication of the results. Yet, the behavioral results are a replication from previous studies on value-based decision-making.
Randomization	There was no group of subject to randomize, but experimental conditions were randomized, as indicated in the methods.
Blinding	There was no group allocation in our design, consequently, there was no need of blinding in our experiments.

Reporting for specific materials, systems and methods

We require information from authors about some types of materials, experimental systems and methods used in many studies. Here, indicate whether each material, system or method listed is relevant to your study. If you are not sure if a list item applies to your research, read the appropriate section before selecting a response.

Materials & experimental systems

n/a	Involved in the study	
☒	☐	Antibodies
☒	☐	Eukaryotic cell lines
☒	☐	Palaeontology and archaeology
☒	☐	Animals and other organisms
☒	☐	Clinical data
☒	☐	Dual use research of concern
☒	☐	Plants

Methods

n/a	Involved in the study	
☒	☐	ChIP-seq
☒	☐	Flow cytometry
☒	☐	MRI-based neuroimaging
